# Detection of Periodontal Pathogens in Oral Samples and Cardiac Specimens in Patients Undergoing Aortic Valve Replacement: A Pilot Study

**DOI:** 10.3390/jcm10173874

**Published:** 2021-08-28

**Authors:** Alessia Pardo, Annarita Signoriello, Caterina Signoretto, Elena Messina, Maria Carelli, Maddalena Tessari, Nunzio Davide De Manna, Cecilia Rossetti, Massimo Albanese, Giorgio Lombardo, Giovanni Battista Luciani

**Affiliations:** 1 Dentistry and Maxillo-Facial Surgery Section, Department of Surgery, Dentistry, Paediatrics and Gynaecology, University of Verona, Piazzale L.A. Scuro 10, 37134 Verona, Italy; alessia.pardo@univr.it (A.P.); annarita.signoriello@univr.it (A.S.); elena.messina26@gmail.com (E.M.); massimo.albanese@univr.it (M.A.); 2 Microbiology Section, Department of Diagnostics and Public Health, University of Verona, Strada Le Grazie 8, 37134 Verona, Italy; caterina.signoretto@univr.it (C.S.); maria.carelli@univr.it (M.C.); 3 Division of Cardiac Surgery, Department of Surgery, Dentistry, Paediatrics and Gynaecology, University of Verona, Piazzale Stefani 1, 37134 Verona, Italy; maddalena.tessari@univr.it (M.T.); d.demanna91@gmail.com (N.D.D.M.); rossetticeci93@gmail.com (C.R.); giovanni.luciani@univr.it (G.B.L.)

**Keywords:** bacteria, cardiovascular, dental plaque, periodontitis, valve

## Abstract

This observational study aimed to: (i) assess the presence of periodontal disease among patients requiring aortic valve replacement; (ii) investigate the presence of oral pathogens in aortic valve specimens and compare them with the microorganisms detected in the oral cavity. Twenty-six patients (15 men and 11 women) were scheduled to be visited the day before the cardiac surgery: periodontal conditions were accurately registered through clinical and radiographic examinations; dental plaque or salivary samples were collected. Valve specimens were collected during surgical aortic valve replacement and analyzed for pathogens detection through microbiological 16SrRna gene sequencing. Bacteria found in plaque samples and valve specimens were assessed according to oral and periodontal conditions. A qualitative comparison between oral and cardiac profiles of the microorganisms detected was performed. The overall number of patients examined for soft tissues conditions was 19, as 7 patients were edentulous. Twelve and three individuals, respectively, presented moderate and severe periodontitis. Nine valves were found to be positive for the presence of oral and periodontopathic bacterial DNA. The microbial species found in valve samples of patients with periodontitis suggest that the presence of these microorganisms in valvular tissue seems to be not coincidental.

## 1. Introduction

The most common dental diseases connected with the presence of oral bacterial species are caries, pulp diseases and periodontitis [1,2]. Recent studies [3] suggested a strong link between infections caused by oral pathogens and several systemic diseases, although detailed responsible mechanisms are still not understood [2]. Among pathological conditions which can be negatively affected by an inflamed oral status, cardiovascular diseases represent a considerable group: major affections comprehend heart valve diseases (regurgitation or stenosis of the aortic or mitral valves) and aortic aneurysms (classified according to the location of the enlarged aorta) [4].

The following hypothetic pathways have been detected to describe the connections between oral infections and secondary systemic effects [5,6]:

- The oral bacteria or their toxins dissemination from the oral cavity into the bloodstream circulation, as a consequence of a transient or prolonged bacteremia (direct injury);
- The inflammation markers and the innate immunity system reaction to the presence of oral microorganisms, which can cause indirectly heart tissue damages (indirect systemic effect).

The first mechanism is mainly connected with professional dental treatments (extraction or subgingival periodontal instrumentation), as well as with usual daily oral care procedures (tooth flossing or chewing) [2,5,7]. The second one is particularly involved in compounded clinical frameworks, such as endodontic infections or chronic periodontitis [5]. The association between periodontitis and cardiovascular diseases has been deeply investigated to identify possible etiological factors in common [2,8,9,10].

Periodontal inflammation is a multifactorial process primarily related to the subgingival biofilm: more than 300 bacterial species constitute a complex community and the onset of periodontitis is linked to a limited number of pathogens in the microbiota, together with a genetic individual predisposition and abundant plaque or calculus deposits. At this proposal, Gram-negative species of the red complex (*Porphyromonas gingivalis*, *Tannerella forsythia*, *Treponema denticola*) were found to assume an important role in releasing virulence factors, such as lipopolysaccharide (LPS), which initiate and perpetuate the production of high levels of proinflammatory cytokines [11]. Matrix metalloproteinases and prostaglandins are, consequently, produced in a cascade reaction [12], leading to a soft tissues inflammation, periodontal attachment loss and alveolar bone resorption. Pathogens finally enter the circulation in great quantities directly from well-vascularized periodontal tissues.

The DNA of periodontal pathogens was found in heart valves, in atrial and myocardial tissues and in atherosclerotic plaque, as shown by several authors [2,10,13,14], suggesting that the entire process of periodontal inflammation contributes to the pathogenesis of cardiovascular diseases. Scientific evidence shows that patients affected by periodontal disease are frequently exposed to the risk of developing bacteremia episodes, which results in high levels of LPS found in the bloodstream [8]. Nevertheless, local or systemic inflammatory changes induced by bacterial presence and their interactions with the host response are currently not completely comprehended.

The present study has three aims: (i) the primary aim is to establish the presence of periodontal disease among patients presenting severe heart valve impairment and requiring aortic valve replacement with eventual coronary bypass surgery; (ii) the secondary aim is to microbiologically feature the presence of periodontal pathogens in aortic valve specimens; (iii) the third aim is to compare oral and cardiac profiles of the microorganisms detected, to hypothesize a possible relationship between them.

## 2. Materials and Methods

### 2.1. Study Design and Inclusion Criteria

An observational study was performed between April and September 2020 at the Cardiovascular Surgery Section, University Hospital of Verona, Verona, Italy. The study protocol was approved by the Ethical Committee of the University of Verona (Protocol n. 21021, approval date 14 April 2020). The study was conducted in accordance with the Ethical Principles of the 64th World Medical Association Declaration of Helsinki and was consistent with good clinical practices. All participants signed a written informed consent.

Subjects referring to the hospital for aortic valve replacement and eventual coronary bypass surgery were enrolled. Inclusion criteria for the study were: (i) age between 35 and 90 years; (ii) acquired heart valve diseases or aortic aneurysms. Exclusion criteria were: (i) pregnant or lactating females; (ii) vulnerable patients, currently undergoing radiotherapy and/or suffering from congenital cardiomyopathies and requiring a cardiac emergency treatment, previously assessed by the surgeon.

### 2.2. Patients’ Examination

Patients enrolled were scheduled to be visited the day before cardiac surgery, to completely assess oral and periodontal conditions and to take plaque or salivary specimens. Antibiotic prophylaxis was administered one hour before dental examination, according to AHA (American Heart Association) guidelines [15], to prevent complications related to the bleeding on probing during both procedures. All dental visits were performed by the same dentist together with the same dental hygienist, at the Periodontology Unit of Dentistry and Maxillo-Facial Surgery Section, University of Verona.

The following variables were assessed during the visit:

- Personal data (sex, age, smoking);
- Health status and clinical history;
- Oral hygiene habits;
- Oral conditions, with the assessment of tooth loss and periodontal evaluation of soft tissues, to establish the presence of periodontal disease; eventual presence of oral ulcerations or neoplasms.

An orthopanoramic radiograph (OPT) had been taken to evaluate the extension of periodontal disease and the degree of bone loss. A dental plaque or salivary sample was collected for the microbiological analysis.

#### 2.2.1. Periodontal Evaluation

Periodontal soft tissues were assessed using a periodontal probe (Florida Probe; Florida Probes Company, Gainesville, FL, USA), applying a force of mild intensity. Six sites on each tooth were explored, three (mesial, central, distal) on the buccal side and three on the lingual/palatal side. The following parameters were collected [16]:

- Probing Pocket Depth (PPD), recorded in mm as the distance between the gingival margin and the base of the periodontal pocket;
- Bleeding on Probing (BOP), recorded as 0 (no bleeding) or 1 (bleeding) after probing for PPD, and expressed in percentage (%) for all sites;
- Visible Plaque Index (VPI), recorded as 0 (no plaque) or 1 (plaque) after probing for PPD, and expressed in % for all sites;
- Clinical Attachment Loss (CAL), recorded in mm as the distance from the cementoenamel junction (CEJ) to the location of the probe tip.

Periodontally healthy conditions were defined as presence of sites with PPD less than 4 mm, even in presence of BOP, and without radiographically detectable bone loss (on the orthopanoramic radiograph). Periodontal impairment was defined, at least in two sites, as a PPD ≥ 4 mm, presence of BOP and radiographically detectable bone loss (on the orthopanoramic radiograph) [16]. Once assessed periodontitis status, its severity was then measured, according to CAL, in moderate (3–4 mm CAL) or severe (≥5 mm CAL) [17].

#### 2.2.2. Plaque Samples and Aortic Valve Specimens

Samples of dental plaque were collected the day before surgery, at the end of periodontal evaluation, as follows: after supra-gingival dental plaque removal, the deepest site was isolated with sterile cotton rolls to properly collect a plaque sample (1 mg ca), through two paper points inserted and left for 30 s at the base of the periodontal pocket. Each collected plaque sample was divided into two Eppendorf tubes: one containing 500 μL of TE buffer (10 mM Tris-HCl, pH 8, 1 mM EDTA) for the molecular investigation by Multiplex PCR and stored at −80 °C until further processing; one containing thioglycolate medium (BD Difco) for cultural investigations. A total of 6 tubes were collected for each patient. In case of edentulous patients, a salivary sample (2 tubes) was taken and stored using the same kind of tubes [18].

Cardiac specimens were collected during the surgical heart valve replacement and stored at −20 °C (RnaLater solution) until analysis execution, through 16SrRna gene sequencing. Type of specimen was the entire aortic valve, precisely composed of two parts: (i) the flaps; (ii) the adjacent part of the aortic root. Furthermore, several aortic valve specimens had calcified cores with circumferential fatty deposits. During the microbiological analysis, the aortic root and a part of valve leaflets were selected and dissected; specimen weight was 25 mg. The main histological tissue in the aortic valve is represented by an endothelial layer, under which there is an intertwining of collagen and elastin fibers [4].

### 2.3. Microbiological Analysis

Molecular biology investigations were conducted by the Microbiology section of the Department of Diagnostics and Public Health, University of Verona. Dental and valve specimens were detected to identify the presence of DNA of different pathogens through PCR end-point and Multiplex PCR.

After excision, valves were immediately placed into a sterile container and transported to the microbiology laboratory within 2 h. Heart valve tissue was stored at −80 until nucleic acids extraction. Each heart valve tissue was manually dissected with sterile scalpels before being placed into a sterile 1.5 mL micro-centrifuge tube. DNA was extracted using the QIAamp DNA Mini Kit protocol for tissues (QIAGEN, Hilden, Germany). Briefly, after re-suspension in 180 μL of digestion Buffer ATL (50 mM Tris HCl, 1 mM EDTA, 0.5% SDS, pH 8.5) and 20 μL of 20 mg/mL Proteinase K solution (QIAGEN), the samples were incubated at 56 °C until total disruption. DNA in the proteolytic digest was further purified according to the manufacturer’s instructions. DNA was eluted in 100 μL of Buffer AE. DNA eluate was stored at −20 °C until use.

Genomic bacterial DNA from plaque was extracted using GenElute™ Bacterial Genomic DNA Kit (Sigma-Aldrich, St. Louis, MO, USA), according to the manufacturer’s instructions, and extract DNA was stored at −20 °C until use.

Two different multiplex PCRs (mPCR) were performed to identify the presence of *P. gingivalis*, *P. intermedia* and *A. actinomycetemcomitans* (mPCR 1) [19], and *T. forsythia*, *T. denticola* and *A. naeslundii* (mPCR 2). Additionally, single PCR was performed to identify *Actinomyces* spp., and *S. mutans.* Primers and PCR conditions are reported in Table S1 (Supplementary Materials). PCR reactions were performed using 5Prime Hot Master Mix (Quantabio, Beverly, MA, USA) according to manufacturer’s instructions.

Valve specimens found positive to the presence of oral bacteria were analyzed through microbiological 16SrRna gene sequencing (BMR Genomics, Padua, Italy) of V3 and V4 portions. A bio-computer analysis was then performed on the valves positive to the genus of possible derivation from the oral cavity, to define the species. The variants of the amplicon sequence (ASV) produced in the 16S-NGS projects (trimming adapters with cutadapt, reads-denoising with DADA2, filtering of the ASVs by frequency (0.005%) and construction of the final feature table) were processed with a QIIME2 plugin called feature-classifier, to associate the taxonomy of Greengenes, Silva and RDP. The same ASVs were then aligned against the Silva 132, RDP and NCBI (nr) databases considering only the best hit. All the taxonomic tables were then merged into a single table, using a Python 3 program specifically developed for this purpose.

### 2.4. Statistical Analysis

Univariate analysis was performed by assessing normality assumptions for quantitative data with the Shapiro–Wilk test; mean and standard deviation (SD) were reported for continuous data that followed a normal distribution; otherwise, median and interquartile range (iqr) were reported; for binary variables (such as VPI, BOP), absolute frequencies, percentages and 95% confidence intervals (C.I.) were reported. The association between categorical variables was tested with χ^2^ test; if any of the expected values was less than 5, a Fisher’s exact test was performed. The comparison between the means of different groups was performed using one-way analysis of variance (ANOVA) or Kruskal–Wallis equality-of-populations rank test. The comparison between the means of 2 different groups (regarding BOP, patients affected by periodontal disease were considered a unique group) was performed using Wilcoxon rank-sum test; a Bonferroni correction for multiple comparison was then applied. Microbiological comparison between plaque samples and aortic valve specimens was performed using McNemar’s test for binary matched-pairs variables. Significance level was set at 0.05 and all analyses were carried out using Stata v.13.0 for Macintosh (StataCorp, College Station, TX, USA).

## 3. Results

### 3.1. Overall Oral Conditions and Presence of Periodontal Disease

Twenty-six patients (15 men and 11 women) attended the study. Demographics are reported in Table 1. One patient referred an episode of previous endocarditis, happened 11 years before the investigation.

**Figure 1:**
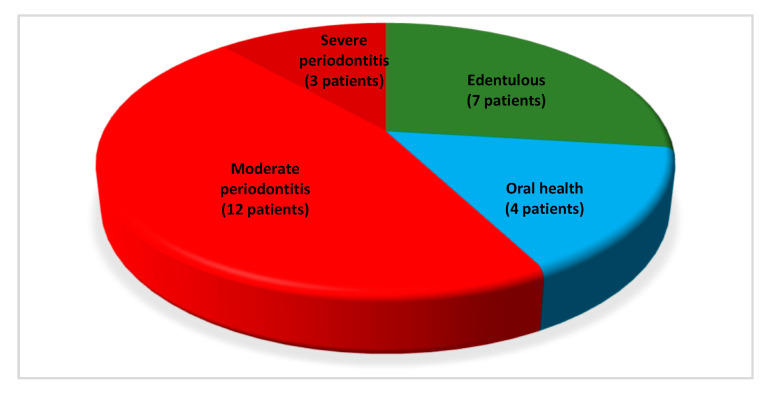
Representation of oral and periodontal conditions in the entire sample.

Out of 26 patients (Figure 1 reports overall oral and periodontal conditions):

- Seven (26.92%) were edentulous and reported to had lost dentition for history of periodontal disease;
- Four dentate patients (15.39%) showed periodontally healthy conditions (oral health);
- Fifteen dentate patients (57.69%) showed periodontitis. Furthermore, the severity of active periodontal disease was registered, with 12 (46.15%) and 3 (11.54%) individuals presenting moderate and severe periodontitis, respectively.

Concerning oral hygiene habits, Table 2 shows a generally poor degree of compliance to daily hygiene procedures and professional oral care. Comparison regarding number of oral hygiene/year and use of interdental devices did not show any statistically significant differences between groups. Furthermore, four and two patients used, respectively, interdental floss and interdental brushes as the type of interdental devices.

None of the patients presented oral ulcerations or neoplasms.

As 7 patients were edentulous, the number of patients examined for periodontal conditions of soft tissues was 19. Table 3 reports the soft tissues assessment and tooth loss for all these 19 patients and according to periodontal conditions: statistically significant differences were found between groups for PPD (*p* = 0.04) and for BOP (*p* = 0.03). The overall number of periodontally compromised sites (PPD ≥ 4 mm, presence of BOP and radiographically detectable bone loss) was 263 in 19 patients. Out of these sites, 152 probed 4 mm, 72 probed 5 mm, 14 probed 6 mm and 24 probed more than 6 mm.

### 3.2. Microbiological Outcomes

Table 4 reports the presence of oral and periodontal bacteria in oral samples, according to oral and periodontal conditions: *Porphyromonas gingivalis*, *Prevotella intermedia*, *Aggregatibacter actinomycetemcomitans*, *Actinomyces naeslundii*, *Tannerella forsythia*, *Treponema denticola* and *Streptococcus mutans* were found. No statistically significant differences in prevalence of these bacteria were found between groups of patients according to oral and periodontal conditions.

NGS analyses showed a bacterial superinfection relevant for the type and number of species; the presence of bacteria was found in 11 out of 26 valve specimens.

In nine out of these eleven specimens, the following oral pathogens were found (Table 5): *Streptococcus periodonticum*, *S. mutans*, *Streptococcus sinensis*, *Streptoccoccus infantis*, *Streptococcus parasanguinis*, *Fusobacterium nucleatum*, *Fusobacterium periodonticum*, *Porphyromonas pasteri* and *Aggregatibacter segnis.* As shown by the species found and differently from the outcomes of the oral samples, no periodontal pathogenic microorganisms belonging to the red complex (*P. gingivalis*, *T. forsythia* and *T. denticola*) were detected in the aortic valve specimens of this study.

Seven out of nine valve specimens positive to oral pathogens were found in patients with periodontal disease (six with moderate and one with severe periodontitis). The absence of oral pathogens was registered in valves of edentulous patients. Nevertheless, no statistically significant differences for the prevalence of bacteria were found between groups of patients according to oral and periodontal conditions.

Finally, comparing similar species, present both in plaque samples and in valve specimens, a statistically significant association (*p* < 0.001) was found for oral *P. gingivalis* and valvular *P. pasteri*; however, this statistical finding has to be considered with caution and from a clinical point of view, as the study sample size was limited.

## 4. Discussion

In the assessment of the primary outcome of the present study, 4 patients were found periodontally healthy, while the majority of patients, 15, showed periodontitis: 12 individuals were affected by moderate and only 3 by severe periodontitis, which is more typical of young individuals (overall mean age in the study was 72 years).

As periodontitis was hypothesized as a risk factor for early atherosclerotic vascular lesions, coronary heart disease, ischemic stroke and myocardial infection [13,20,21,22,23], an accurate evaluation of periodontal conditions among patients affected by these conditions is recommended [9,21,23]. Regarding the accumulation of dental plaque, often related to periodontitis [24], a mean VPI of 22% was registered, and slightly more than half of patients showed sufficient plaque control, demonstrating a general poor compliance to oral health maintenance. These outcomes are comparable to other investigations in literature with the same type of patients [25,26].

Concerning secondary outcomes of the study, microbiological profiles of oral samples and aortic valve specimens were described and compared, to verify possible similarities in terms of bacterial species [27], hypothesizing an association between dental health and cardiovascular disease. Our findings regarding the presence of oral pathogens both in plaque samples and in cardiac specimens can be generally confirmed by similar results in the literature [9,14,28]. It is necessary to underline that seven out of nine valve specimens positive to oral pathogens were present in patients with periodontal disease (six with moderate and one with severe periodontitis), evidencing the possible role of plaque deposits as a reservoir of bacteria capable of entering the circulation [29,30]. No statistically significant differences for the presence of oral bacteria in valve specimens were found between patients with different oral and periodontal conditions. Despite that, comparing similar species present both in plaque samples and in valve specimens, a statistically significant association was found for *P. gingivalis* and *P. pasteri* in the oral sample and in the valve specimen, respectively. From a clinical point of view, this result suggests that oral bacteria found in aortic valve samples, associated or not with periodontal disease, may have a direct or indirect role in the development of cardiovascular disease.

Furthermore, as many studies identified periodontal pathogens in atherosclerotic samples [31,32,33,34,35], it seems that an increased risk for developing atherosclerosis in people with periodontitis can be significantly postulated. At this proposal, a specific pathogen (e.g., *P. gingivalis*), influencing lipoprotein metabolism and promoting inflammatory responses in sites distant from the oral cavity, may be involved in atherosclerosis [36,37,38].

Nevertheless, other authors declared different outcomes, establishing an unclear association between periodontitis and cardiovascular diseases [10,21,39,40], especially considering that the link assumes evidence only according to the same phylogenetic origin for the two body districts [41].

Raffaelli et al. [10] stated that, despite a highly effective PCR analysis for small specimens, a high blood pressure at the aortic valve (situated between the aorta and left ventricle) may prevent the adhesion and consequent proliferation of bacteria; moreover, an indirect effect could be played instead by the inflammation markers and the immune system in response to the present bacteria, which finally do not cause a direct injury. This hypothesis seems to be valid, as it is not possible to exactly establish a persistent or transient condition derived from the bacterial colonization of atheromatous plaques, whose oxidative status represents an ideal condition for anaerobic bacteria [32].

Transient bacteremia episodes consequent to oral hygiene procedures, especially in periodontal patients, are easily limited by defense mechanisms usually present in periodontally healthy patients [42]. On the other hand, the systematic removal of biofilm is essential in individuals with no efficient reactions of the immune system, e.g., patients considered in this investigation [31,43]. In our study, one patient referred to an episode of previous endocarditis. At this regard, some pathogens found in the study were also related in literature to cases of infective endocarditis: *S. parasanguinis*, associated with the *viridans* group [44], *S. sinensis* [45] and *A. segnis* [46].

Some authors [9,14] described biochemical, histological and immunohistochemical aspects to better understand how the link between periodontal disease and cardiovascular disease can be connected to the immune response. Despite an evident association between inflammation scores and periodontal indexes, the detection of LPS-binding protein in cardiac tissues did not unequivocally confirm a direct link between periodontal conditions and cardiovascular disease [9].

Referring to the mechanisms underlying the chronic inflammatory process of atherosclerosis, the individual role of oral bacteria in promoting it was outlined [14]: the fimbria of *P. gingivalis* has the capacity to adhere to the endothelium and to invade it; a phenotype of *A. actinomycetemcomitans* can invade the endothelial cells via the receptor for the platelet-activating factor; thus, gaining accessing to the systemic circulation. At this proposal, the function of some pathogens specifically found in the present study need to be deepened, as it was only partially assessed in the literature: *S. periodonticum*, oral pathogen isolated in the plaque of periodontal lesions [47]; *F. periodonticum*, associated with the orange complex [48]; *P. pasteri*, associated with *P. gingivalis* [48,49].

Besides specific periodontal microorganisms, also other bacteria species (oral streptococcal) were detected in this study, as confirmed by other investigations [5], in which *S. mutans* was found as the most frequently detected species in valve specimens [1,2].

Up-to-date literature does not offer extended results about the prevalence of periodontitis in cardiovascular patients, moreover cardiovascular affections classified are numerous [4] and this categorization makes it difficult to be accurate in identifying patients to analyze. The present study was, thus, pioneering and aimed to constitute an initial overview of periodontal conditions among patients with severe conditions of aortic valve impairment. We are aware that the small number of patients assessed in the study represents a strong limitation and it would be advisable to enlarge the sample to obtain more sound results. Difficulties in these terms are evident, as patients with cardiovascular disorders frequently present also other systemic comorbidities (e.g., diabetes and hypertensions), which could represent a high risk for undergoing surgery: a multicentric approach could be advisable in terms of an easier enrolment of patients. Furthermore, NGS analyses surprisingly highlighted a bacterial superinfection relevant for the type and number of species: taking into account the standards adopted to reduce the risk of contamination in valves sampling, authors suggest that these data are worthy of attention.

A strength of the present study could otherwise be represented by the specific assessment of periodontal conditions, with 12 and 3 individuals presenting moderate and severe periodontitis; moreover, an overall number of periodontally compromised sites of 263 was found in 19 out of 26 patients. As only seven patients were compliant to a strict maintenance program of oral hygiene, authors strongly support the need of a reinforced periodontal care, essential for supporting oral health and, consequently, heart conditions [50] in patients undergoing aortic valve replacement and eventual coronary bypass surgery.

## 5. Conclusions

No periodontal pathogenic microorganisms belonging to the red complex were detected in aortic valve specimens of this study. However, the significant number of similar oral bacterial DNA species found in cardiac samples of patients with periodontitis, compared to edentulous patients, suggests that the presence of these microorganisms is associated with the way of a direct mechanism, due to the increased vascular bed consequent to the inflammation typical of periodontal pockets. As the presence of these bacteria in valve tissue seems to be not coincidental, we hypothesized that they may have a role in the development of cardiovascular diseases.

Further multicentric studies, with a larger sample size, are desirable to clarify the hypothesis which supports a direct association between periodontitis and cardiovascular disease, and to define if periodontal care can play a crucial role in supporting the oral conditions of patients with cardiac impairment.

## Figures and Tables

**Table 1:** Demographics. Variables related to patients are expressed as *n* (%); age is presented as mean ± standard deviation (SD).

Variable	*n* (%)
**Sex**	
male	15 (57.69)
female	11 (42.31)
**Age**	72 ± 10 years
**Smoking**	
no	24 (92.31)
yes (>25 cigarettes/day)	2 (7.69)
**Cardiovascular disease**	
aortic valve regurgitation	3 (11.54)
severe aortic stenosis	17 (65.38)
severe aortic stenosis + coronopathy	6 (23.08)
**Previous endocarditis episodes**	
no	25 (96.15)
yes	1 (3.85)
**Antiplatelet therapy**	
no	12 (46.15)
yes	14 (53.85)
**Anticoagulant therapy**	
no	23 (88.46)
yes	3 (11.54)
**Type of oral examination**	
edentulous patient	7 (26.92)
tooth site	19 (73.08)
**Type of oral sample**	
saliva (edentulous patient)	6 (23.08)
plaque (tooth site)	10 (38.46)
saliva + plaque (edentulous patient)	10 (38.46)

**Table 2:** Oral hygiene habits; values are expressed as *n* (%).

	Overall	Oral and Periodontal Conditions				
		Oral Health *n* (%)	Moderate Periodontitis *n* (%)	Severe Periodontitis *n* (%)	Edentulousn (%)	*p* Value
Oral professional hygiene/year						
not regular	4 (15.39)	0 (0.00)	2 (16.67)	0 (0.00)	2 (28.57)	0.62
1/year	15 (57.69)	3 (75.00)	5 (41.67)	3 (100.00)	4 (57.14)	
2/year	7 (26.92)	1 (25.00)	5 (41.67)	0 0.00)	1 (14.29)	
Use of daily interproximal oral hygiene devices						
no	20 (76.92)	3 (75.00)	8 (66.67)	2 (66.67)	7 (100.00)	0.34
yes	6 (23.08)	1 (25.00)	4 (33.33)	1 (33.33)	0 (0.00)	

**Table 3:** Variables related to soft tissues and tooth loss (overall and according to periodontal conditions) of 19 dentate patients.

	Overall	Periodontal Conditions			
		Oral Health	Moderate Periodontitis	Severe Periodontitis	*p* Value
PPD (mm)	4.2 (0.5)	4.2 (0.25)	4.2 (0.2)	4.6 (1.4)	0.04 *
BOP (%)	14 (12)	4 (3)	17 (11)	20 (17)	0.03 *
VPI (%)	22 (43)	33 (61)	21 (52)	56 (34)	0.52
CAL (mm)	4.4 (0.6)	4.4 (0.4)	4.3 (0.4)	5 (1)	0.051
Tooth loss	12 (7)	10 (3)	11 (7)	14 (10)	0.77

Variables are expressed as median (iqr, interquartile range); PPD and CAL are expressed in (mm); BOP and VPI are expressed in (%); *, statistically significant; Bonferroni correction is reported for BOP.

**Table 4:** Presence of oral and periodontal bacteria in oral samples (overall and according to oral and periodontal conditions); values are expressed as *n* (%).

Oral Samples	Overall	Oral and Periodontal Conditions				
	*n* (%)	Oral Health *n* (%)	Moderate Periodontitis *n* (%)	Severe Periodontitis *n* (%)	Edentulous *n* (%)	*p* Value
*P. gingivalis*						
no	5 (19.23)	1 (25.00)	1 (8.33)	0 (0.00)	3 (42.86)	
yes	21 (80.77)	3 (75.00)	11 (91.67)	3 (100.00)	4 (57.14)	0.28
*P. intermedia*						
no	0 (0.00)	0 (0.00)	0 (0.00)	0 (0.00)	0 (0.00)	
yes	26 (100.00)	4 (100.00)	12 (100.00)	3 (100.00)	7 (100.00)	/
*A. actinomycetemcomitans*						
no	0 (0.00)	0 (0.00)	0 (0.00)	0 (0.00)	0 (0.00)	
yes	26 (100.00)	4 (100.00)	12 (100.00)	3 (100.00)	7 (100.00)	/
*T. denticola*						
no	21 (80.77)	3 (75.00)	9 (75.00)	2 (66.67)	7 (100.00)	
yes	5 (19.23)	1 (25.00)	3 (25.00)	1 (33.33)	0 (0.00)	0.39
*A. naeslundii*						
no	10 (38.46)	1 (25.00)	4 (33.33)	0 (0.00)	5 (71.43)	
yes	16 (61.54)	3 (75.00)	8 (66.67)	3 (100.00)	2 (28.57)	0.18
*S. mutans*						
no	20 (76.92)	2 (50.00)	9 (75.00)	3 (100.00)	6 (85.71)	
yes	6 (23.08)	2 (50.00)	3 (25.00)	0 (0.00)	1 (14.29)	0.55
*T. forsythia*						
no	9 (34.62)	1 (25.00)	3 (25.00)	1 (33.33)	4 (57.14)	
yes	17 (65.38)	3 (75.00)	9 (75.00)	2 (66.67)	3 (42.86)	0.59

**Table 5:** Presence of oral bacteria in valve specimens (overall and according to oral and periodontal conditions); values are expressed as *n* (%).

Valve Specimens	Overall	Oral and Periodontal Conditions				
	*n* (%)	Oral Health *n* (%)	Moderate Periodontitis *n* (%)	Severe Periodontitis *n* (%)	Edentulous *n* (%)	*p* Value
*S. periodonticum*						
no	22 (84.62)	2 (50.00)	11 (91.67)	2 (66.67)	7 (100.00)	
yes	4 (15.38)	2 (50.00)	1 (8.33)	1 (33.33)	0 (0.00)	0.09
*S. mutans*						
no	23 (88.46)	4 (100.00)	9 (75.00)	3 (100.00)	7 (100.00)	
yes	3 (11.54)	0 (0.00)	3 (25.00)	0 (0.00)	0 (0.00)	0.39
*S. sinensis*						
no	24 (92.31)	4 (100.00)	11 (91.67)	2 (66.67)	7 (100.00)	
yes	2 (7.69)	0 (0.00)	1 (8.33)	1 (33.33)	0 (0.00)	0.39
*S. infantis*						
no	25 (96.15)	3 (75.00)	12 (100.00)	3 (100.00)	7 (100.00)	
yes	1 (3.85)	1 (25.00)	0 (0.00)	0 (0.00)	0 (0.00)	0.26
*S. parasanguinis*						
no	25 (96.15)	4 (100.00)	12 (100.00)	2 (66.67)	7 (100.00)	
yes	1 (3.85)	0 (0.00)	0 (0.00)	1 (33.33)	0 (0.00)	0.11
*F. nucleatum*						
no	25 (96.15)	4 (100.00)	11 (91.67)	3 (100.00)	7 (100.00)	
yes	1 (3.85)	0 (0.00)	1 (8.33)	0 (0.00)	0 (0.00)	0.75
*F. periodonticum*						
no	25 (96.15)	4 (100.00)	12 (100.00)	2 (66.67)	7 (100.00)	
yes	1 (3.85)	0 (0.00)	0 (0.00)	1 (33.33)	0 (0.00)	0.11
*P. pasteri*						
no	25 (96.15)	4 (100.00)	12 (100.00)	2 (66.67)	7 (100.00)	
yes	1 (3.85)	0 (0.00)	0 (0.00)	1 (33.33)	0 (0.00)	0.11
*A. segnis*						
no	25 (96.15)	4 (100.00)	11 (91.67)	3 (100.00)	7 (100.00)	
yes	1 (3.85)	0 (0.00)	1 (8.33)	0 (0.00)	0 (0.00)	0.75

## Data Availability

The data presented in this study are available on request from the corresponding author.

## Supplementary Materials

The following are available online at https://www.mdpi.com/article/10.3390/jcm10173874/s1, Table S1: primers and PCR conditions used in the study.

## Abbreviations

LPS	Lipopolysaccharide
AHA	American Heart Association
OPT	Orthopanoramic radiograph
PPD	Probing Pocket Depth
BOP	Bleeding on Probing
VPI	Visible Plaque Index
CAL	Clinical Attachment Loss
CEJ	Cementoenamel junction
PCR	Polymerase Chain Reaction
SD	Standard deviation
iqr	Interquartile range
ANOVA	One-way analysis of variance
*P. gingivalis*	*Porphyromonas gingivalis*
*P. intermedia*	*Prevotella intermedia*
*T. denticola*	*Treponema denticola*
*T. forsythia*	*Tannerella forsythia*
*A. actinomycetemcomitans*	*Aggregatibacter actinomycetemcomitans*
*A. naeslundii*	*Actinomyces naeslundii*
*S. mutans*	*Streptococcus mutans*
*S. periodonticum*	*Streptococcus periodonticum*
*S. sinensis*	*Streptococcus sinensis*
*S. infantis*	*Streptoccoccus infantis*
*S. parasanguinis*	*Streptococcus parasanguinis*
*F. nucleatum*	*Fusobacterium nucleatum*
*F. periodonticum*	*Fusobacterium periodonticum*
*P. pasteri*	*Porphyromonas pasteri*
*A. segnis*	*Aggregatibacter segnis*
