# Supplementary material for: Detection of Periodontal Pathogens in Oral Samples and Cardiac Specimens in Patients Undergoing Aortic Valve Replacement: A Pilot Study
Source: J Clin Med. 2021 Aug 28;10(17):3874. doi: 10.3390/jcm10173874 (PMC8432007; doi:10.3390/jcm10173874)
Supplement: Supplementary file 1 [file jcm-10-03874-s001.zip › jcm-1332175-supplementary.pdf]

Journal of
Clinical Medicine

Supplementary Material

Table S1. Primers and PCR conditions used in the study.

Type	Target	Sequence	Reference	PCR	Annealing
F	16 s rRNA	CGT GCC AGC AGC CGC GGT AAT ACG	Garcia et al., 2013 [1]	mPCR1	70 °C
R	PI	TCC GCA TAC GTT GCG TGC ACT CAA G			
R	PG	TAC ATA GAA GCC CCG AAG GAA GAC G			
R	AG	CTT TGC ACA TCA GCG TCA GTA CAT CCC CAA GG			
F	TD	GCA AGA CTT GTA GCG GTA GT	This study	mPCR2	60 °C
R	TD	GAT GCC TAT TTG CGG GCT TG			
F	TF	CGG TGG TCT CCA ATC TCA CC			
R	TF	GCC CTC AAC ACA CGA CAC TT			
F	AN	GGA ATG ATG GCG TGA ATG GC			
R	AN	CCG ATC CCG TGA GTA CAT GG			
F	AC	GGC KTG CGG TGG GTA CGGG C	Xia et al., 2003 [2]	sPCR	60 °C
R	AC	GGC TTT AAG GGA TTC GCT CCR CCT CAC			
F	SM	GGCACCACAACATTGGGAAGCTCAGTT	Nakano et al., 2006 [3]	sPCR	70 °C
R	SM	GGAATGGCCGCTAAGTCAACAGGAT			

Abbreviations: F = forward primer; R = reverse primer; PG = *P. gingivalis*; PI = *P. intermedia*; AG = *A. actinomycetemcomitans*; AN = *A. naeslundii*; TF = *T. forsythia*; TD = *T. denticola*; AC = *Actinomyces* spp.; SM = *S. mutans*. mPCR1 = multiple PCR 1; mPCR2 = multiple PCR 2; sPCR = single PCR.

References

1. García, L.; Tercero, J.C.; Legido, B.; Ramos, J.A.; Alemany, J.; Sanz, M. Rapid detection of *Actinobacillus actinomycetemcomitans, Prevotella intermedia* and *Porphyromonas gingivalis* by multiplex PCR. *J Periodontal Res* **1998**, *33*, 59-64. doi: 10.1111/j.1600-0765.1998.tb02292.x.
2. Xia, T.; Baumgartner, J.C. Occurrence of Actinomyces in Infections of Endodontic Origin. *J. Endod.* **2003**, *29*, 549–552, doi:10.1097/00004770-200309000-00001.
3. Nakano, K.; Inaba, H.; Nomura, R.; Nemoto, H.; Takeda, M.; Yoshioka, H.; Matsue, H.; Takahashi, T.; Taniguchi, K.; Amano, A.; et al. Detection of cariogenic Streptococcus mutans in extirpated heart valve and atheromatous plaque specimens. *J. Clin. Microbiol.* **2006**, *44*, 3313–3317, doi:10.1128/JCM.00377-06.
